# Congenital Afibrinogenemia With Coexisting Factor V Leiden Mutation Complicated by Budd-Chiari Syndrome: A Case Report

**DOI:** 10.7759/cureus.97055

**Published:** 2025-11-17

**Authors:** Rim A Boutari, Fatima I Hsayan, Fatmeh Mallah, Moustafa Diab

**Affiliations:** 1 Gastroenterology and Hepatology, Faculty of Medicine, Lebanese University, Beirut, LBN; 2 Radiology, Faculty of Medicine, Lebanese University, Beirut, LBN; 3 Gastroenterology and Hepatology, Al Zahraa Hospital University Medical Center, Beirut, LBN

**Keywords:** bleeding and thrombosis, budd-chiari syndrome, congenital afibrinogenemia, factor v leiden mutation, hepatic venous outflow obstruction, thrombophilia

## Abstract

Congenital afibrinogenemia is a rare autosomal recessive bleeding disorder characterized by the complete absence of fibrinogen, a key protein in the coagulation cascade. Patients usually present with hemorrhagic manifestations, ranging from mild mucocutaneous bleeding to life-threatening events, such as intracranial or gastrointestinal hemorrhage. Paradoxically, thrombotic complications have been increasingly described, underscoring the dual nature of the disease. We report a case of a 15-year-old female with congenital afibrinogenemia who presented with abdominal pain and was diagnosed with Budd-Chiari syndrome (BCS), a rare but potentially fatal hepatic venous outflow obstruction. Genetic testing further revealed the presence of a heterozygous factor V Leiden mutation, a well-known inherited thrombophilia that likely contributed to the development of BCS in this patient. She was treated with fibrinogen replacement and anticoagulation therapy, with careful monitoring to balance bleeding and thrombotic risks. The patient showed clinical improvement and remained stable at three-month follow-up. The coexistence of a bleeding disorder and a prothrombotic mutation illustrates the complexity of clinical management, as therapeutic strategies must balance hemostatic replacement with anticoagulation while minimizing risks on both sides. This case highlights the need for heightened awareness of thrombotic complications in congenital afibrinogenemia, the importance of investigating concomitant thrombophilic risk factors, and the necessity of an individualized, multidisciplinary approach to optimize outcomes in rare coagulation disorders.

## Introduction

Congenital afibrinogenemia is an extremely rare autosomal recessive bleeding disorder characterized by the complete absence or undetectable levels of fibrinogen, a critical protein involved in clot formation, platelet aggregation, and wound healing. Its estimated global prevalence is less than one per million, with most cases identified in neonates presenting with umbilical stump bleeding, followed later by recurrent mucocutaneous hemorrhage, hemarthroses, or menorrhagia [1,2]. The condition results from mutations in one of the three fibrinogen genes - fibrinogen alpha (FGA) chain, beta (FGB) chain, or gamma (FGG) chain - which impair fibrinogen synthesis, assembly, or secretion [3].

Although bleeding manifestations dominate the clinical spectrum, paradoxical thrombotic events have been increasingly recognized in patients with afibrinogenemia. Documented complications include deep vein thrombosis, pulmonary embolism, and, more rarely, Budd-Chiari syndrome (BCS) [4]. BCS is defined as hepatic venous outflow obstruction, which can result in hepatomegaly, ascites, abdominal pain, and portal hypertension. It is most often associated with myeloproliferative disorders or inherited thrombophilia; however, its occurrence in patients with congenital bleeding disorders is both unexpected and poorly understood [5].

The presence of additional prothrombotic mutations, such as factor V Leiden, may further exacerbate thrombotic risk in this population. Factor V Leiden is the most common inherited thrombophilia, and even in the heterozygous state, it significantly increases the risk of venous thromboembolism, particularly when combined with other clinical or genetic risk factors [6]. The coexistence of congenital afibrinogenemia and factor V Leiden is exceptionally rare, but when present, it creates a highly complex clinical scenario in which patients are simultaneously predisposed to severe bleeding and paradoxical thrombosis.

In this report, we present a case of a 15-year-old female with congenital afibrinogenemia complicated by Budd-Chiari syndrome in the context of heterozygous factor V Leiden mutation. This case highlights the dual hemostatic risks inherent to such patients and underscores the challenges in therapeutic decision-making, requiring careful, multidisciplinary management.

## Case presentation

A 15-year-old female with a known history of congenital afibrinogenemia presented to the emergency department with progressive abdominal distension and dull, non-radiating pelvic pain for two weeks. She reported regular menstrual cycles, with her last menses occurring two weeks prior to presentation, and a urine pregnancy test was negative. She denied fever, nausea, vomiting, gastrointestinal bleeding, or changes in bowel habits. Her bleeding disorder had been managed intermittently with fibrinogen concentrate (Haemocomplettan), with no recent adjustments to her regimen.

On examination, she was afebrile and hemodynamically stable. She appeared pale and fatigued, with mild temporal wasting and reduced subcutaneous fat, consistent with chronic illness. Abdominal assessment revealed distension, hepatomegaly, and shifting dullness, consistent with ascites. There were no stigmata of chronic liver disease, such as spider angiomas or palmar erythema.

Initial laboratory investigations revealed a hemoglobin level of 11.8 g/dL, platelet count of 315×10⁹/L, and serum creatinine of 0.6 mg/dL. Liver function tests were within normal limits, including aspartate aminotransferase (AST: 18.8 U/L), alanine aminotransferase (ALT: 14.1 U/L), and alkaline phosphatase (71 U/L). Total bilirubin was 0.88 mg/dL, with a direct fraction of 0.35 mg/dL. Lactate dehydrogenase (LDH) was mildly reduced at 102 U/L, and serum albumin was preserved at 41.2 g/L. Notably, the international normalized ratio (INR) was markedly elevated (>8), reflecting impaired coagulation consistent with her underlying afibrinogenemia. These findings are summarized in Table 1.

**Table 1:** Initial laboratory results of the patient. AST: aspartate aminotransferase; ALT: alanine aminotransferase; Alk phos: alkaline phosphatase; LDH: lactate dehydrogenase; INR: international normalized ratio

Parameters	Value	Normal range
Hemoglobin	11.8 g/dL	12-16 g/dL
Platelets	315×10⁹/L	150-450×10⁹/L
Creatinine	0.6 mg/dL	0.6-1.3 mg/dL
AST	18.8 U/L	10-40 U/L
ALT	14.1 U/L	7-56 U/L
Alk phos	71 U/L	44-147 U/L
Total bilirubin	0.88 mg/dL	0.1-1.2 mg/dL
Direct bilirubin	0.35 mg/dL	0.0-0.3 mg/dL
LDH	102 U/L	140-280 U/L
Albumin	4.12 g/L	3.5-5.0 g/L
INR	>8	0.8-1.2

Given the patient’s clinical presentation and the timing of evaluation (off-hours), Doppler ultrasound was not performed initially. Instead, a contrast-enhanced abdominal CT scan was obtained due to its immediate availability and broader diagnostic scope, allowing simultaneous assessment of hepatic vasculature, parenchymal changes, and ascites.

**Figure 1:**
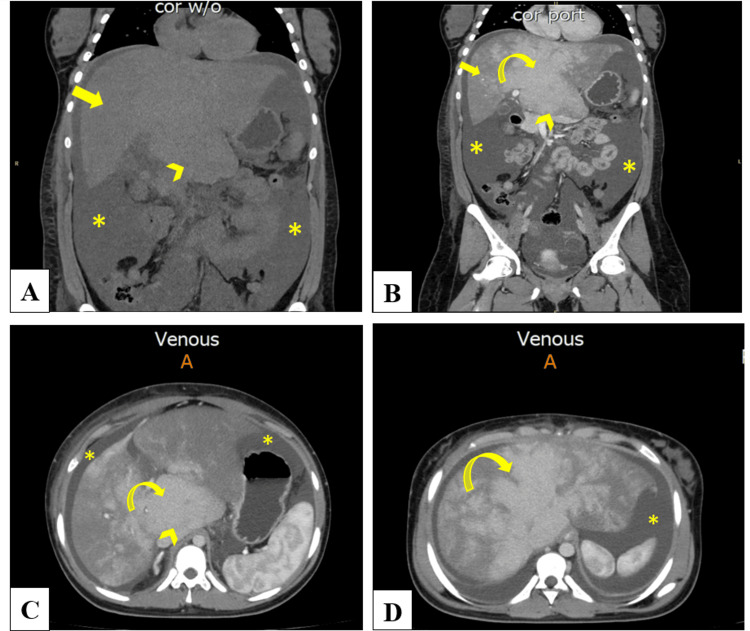
Contrast-enhanced CT showing findings characteristic of Budd-Chiari syndrome. (A) Non-contrast coronal CT showing hepatomegaly (arrow), caudate lobe hypertrophy (arrowhead), and ascites (asterisk). (B) Coronal portal venous phase with contrast showing hepatomegaly (arrow), caudate lobe hypertrophy (arrowhead), ascites (asterisk), and central-predominant enhancement with peripheral hypoenhancement (curved arrow), consistent with hepatic venous outflow obstruction. (C, D) Axial portal venous phase images showing caudate lobe hypertrophy (arrowhead), ascites (asterisk), and a mottled “nutmeg” liver appearance (curved arrow) reflecting centrilobular congestion. These combined findings - hepatomegaly, ascites, caudate lobe hypertrophy, central enhancement, and “nutmeg” pattern - are characteristic of Budd-Chiari syndrome.

A contrast-enhanced abdominal CT scan revealed hepatomegaly with caudate and left lobe hypertrophy, heterogeneous hepatic parenchymal enhancement, and moderate-to-large ascites. The right hepatic lobe measured approximately 18.7 cm in craniocaudal span (normal: <15 cm), and the caudate lobe measured 4.8 cm in transverse diameter (normal: <3.5 cm), confirming radiologic hepatomegaly. Hepatic veins were not visualized, suggestive of hepatic venous outflow obstruction. Representative imaging findings are shown in Figure 1A-1D.

Diagnostic paracentesis yielded clear yellow fluid with a serum-ascites albumin gradient (SAAG) of 1.54 g/dL, consistent with portal hypertension. In accordance with current guidelines recommending comprehensive evaluation for Budd-Chiari syndrome, even when a known risk factor exists, genetic testing was performed and revealed heterozygosity for the factor V Leiden mutation, an inherited thrombophilia likely contributing to thrombosis in this patient.

Given the dual risk of bleeding and thrombosis, she was managed with carefully balanced therapy as follows: fibrinogen replacement (Haemocomplettan) to correct coagulopathy, alongside anticoagulation to treat BCS. She was initiated on oral rivaroxaban (Xarelto) at a dose of 20 mg once daily. Diuretics were initiated for symptomatic ascites, consisting of oral furosemide (Lasix) 40 mg once daily and spironolactone (Aldactone) 100 mg once daily. Fibrinogen levels were maintained above 1.0 g/L using intermittent Haemocomplettan infusions to support hemostasis during anticoagulation, with close monitoring of clinical status and bleeding signs.

At three-month follow-up, the patient remained clinically stable, with reduction in abdominal girth, resolution of ascites on imaging, and no bleeding or further thrombotic events. She continued oral rivaroxaban (Xarelto) 20 mg once daily beyond the initial three-month period, with intermittent Haemocomplettan infusions as needed to maintain fibrinogen levels above 1.0 g/L. Regular outpatient follow-up was maintained, and no adverse events were reported during the extended monitoring period.

## Discussion

This case illustrates the paradoxical development of Budd-Chiari syndrome (BCS), a thrombotic complication, in a patient with congenital afibrinogenemia, a condition traditionally associated with bleeding. While afibrinogenemia typically presents with spontaneous hemorrhage, recent evidence suggests that patients may also be predisposed to thrombosis due to unopposed thrombin activity in the absence of fibrin, which normally serves as a negative feedback regulator within the coagulation cascade [4,7].

Thrombotic events in afibrinogenemia may occur spontaneously or following fibrinogen replacement therapy, particularly when administered at high doses or frequent intervals [8]. In this patient, intermittent administration of Haemocomplettan may have transiently amplified thrombin generation, contributing to hepatic venous thrombosis. The coexistence of heterozygous factor V Leiden mutation, a common inherited thrombophilia, further compounded the thrombotic risk, highlighting the additive effect of multiple prothrombotic factors in the context of a congenital bleeding disorder.

In accordance with the latest European Association for the Study of the Liver (EASL) Clinical Practice Guidelines, a comprehensive thrombophilia workup is recommended for all patients presenting with BCS, even when a known predisposing condition exists, to optimize risk stratification and guide therapy [9].

Diagnosis of BCS requires a high index of suspicion and appropriate imaging. Doppler ultrasound, computed tomography (CT), or magnetic resonance imaging (MRI) may reveal hepatic vein obstruction, caudate lobe hypertrophy, and ascites, hallmark features of hepatic venous outflow obstruction [5]. In this case, contrast-enhanced CT demonstrated classic findings, which were supported by paracentesis showing a high serum-ascites albumin gradient (SAAG), consistent with portal hypertension.

Management of thrombosis in afibrinogenemia poses a unique challenge due to the dual risk of bleeding and thrombosis. While anticoagulation is the cornerstone of BCS treatment, it may be contraindicated in patients with severe coagulopathy. In this case, a carefully balanced approach was adopted, combining fibrinogen replacement to correct coagulopathy with anticoagulation to address hepatic venous thrombosis, under close clinical monitoring. This strategy enabled safe and effective management of the thrombotic event while minimizing bleeding risk. Similar cases in the literature have reported favorable outcomes with tailored therapy when anticoagulation is considered high-risk [10].

For patients with severe or recurrent BCS, liver transplantation remains a definitive treatment option, resolving venous obstruction and restoring endogenous fibrinogen production [11]. This case underscores the importance of individualized, multidisciplinary management in patients with congenital bleeding disorders complicated by thrombosis and reinforces the need to screen for additional prothrombotic risk factors, even when one established risk factor is present.

## Conclusions

This case underscores a rare but clinically significant occurrence of Budd-Chiari syndrome in a patient with congenital afibrinogenemia, illustrating the paradoxical coexistence of bleeding and thrombotic risks in congenital coagulation disorders. It highlights the importance of comprehensive evaluation for additional prothrombotic risk factors, even when a known bleeding disorder is present, in accordance with current guidelines. Optimal management requires a carefully balanced, individualized therapeutic approach, combining hemostatic replacement with anticoagulation when feasible, alongside close clinical monitoring. Multidisciplinary collaboration among hematology, hepatology, and gastroenterology specialists is crucial to navigate the complexities of such cases and to maximize patient safety and outcomes. Further research is needed to better define risk stratification and long-term management strategies for thrombotic events in patients with rare bleeding disorders.

## References

[1] de Moerloose P, Neerman-Arbez M (2009). Congenital fibrinogen disorders. Semin Thromb Hemost.

[2] Zhang Y, Zuo X, Teng Y (2020). Women with congenital hypofibrinogenemia/afibrinogenemia: from birth to death. Clin Appl Thromb Hemost.

[3] Casini A, de Moerloose P, Neerman-Arbez M (2016). Clinical features and management of congenital fibrinogen deficiencies. Semin Thromb Hemost.

[4] Casini A, Neerman-Arbez M, de Moerloose P (2021). Heterogeneity of congenital afibrinogenemia, from epidemiology to clinical consequences and management. Blood Rev.

[5] Plessier A, Valla DC (2008). Budd-Chiari syndrome. Semin Liver Dis.

[6] Ageno W, Squizzato A, Garcia D, Imberti D (2006). Epidemiology and risk factors of venous thromboembolism. Semin Thromb Hemost.

[7] Nikam VG, Dhakre VW, Motwani K, Chattopadhyay S (2024). Budd-Chiari syndrome associated with congenital afibrinogenaemia reversed after orthotopic liver transplant. BMJ Case Rep.

[8] Buitrago L, Langdon WY, Sanjay A, Kunapuli SP (2011). Tyrosine phosphorylated c-Cbl regulates platelet functional responses mediated by outside-in signaling. Blood.

[9] (2016). EASL Clinical Practice Guidelines: vascular diseases of the liver. J Hepatol.

[10] Acharya SS (2013). Rare bleeding disorders in children: identification and primary care management. Pediatrics.

[11] Gallastegui N, Kimble EL, Harrington TJ (2016). Resolution of fibrinogen deficiency in a patient with congenital afibrinogenemia after liver transplantation. Haemophilia.

